# Work Ability among Italian Bank Video Display Terminal Operators: Socio-Demographic, Lifestyle, and Occupational Correlates

**DOI:** 10.3390/ijerph16091653

**Published:** 2019-05-12

**Authors:** Giacomo Garzaro, Ilaria Sottimano, Matteo Di Maso, Enrico Bergamaschi, Maurizio Coggiola, Daniela Converso, Sergio Iavicoli, Enrico Pira, Sara Viotti

**Affiliations:** 1 Department of Public Health and Pediatrics, University of Turin, P.zza Polonia 94, 10126 Turin, Italy; giacomo.garzaro@unito.it (G.G.); matteo.dimaso@unito.it (M.D.M.); enrico.bergamaschi@unito.it (E.B.); maurizio.coggiola@unito.it (M.C.); enrico.pira@unito.it (E.P.); 2 Department of Psychology, University of Turin, via Verdi 10, 10124 Turin, Italy; daniela.converso@unito.it (D.C.); sara.viotti@unito.it (S.V.); 3 National Institute for Insurance against Accidents at Work, P.le Pastore 6, 00144 Rome, Italy; s.iavicoli@inail.it

**Keywords:** work ability, video display terminal operators, aging, lifestyle, occupational health, cross-sectional study, banking sector

## Abstract

Bank employees, especially video display terminal (VDT) operators, are constantly exposed to various occupational risks, such as the adoption of awkward postures, repetitive finger movements, and utilization of software with poor usability, which may lead to computer visual syndrome, tension headache, lower back pain, and/or stress, which compromises their overall health and work ability (WA). Thus, in this cross-sectional study, we aimed to establish that the determinants among socio-demographic, lifestyle, and occupational characteristics are associated with impaired WA in bank VDT operators. To this end, we administered a set of socio-demographic, lifestyle, occupational, and Work Ability Index (WAI) questionnaires to 2077 Italian bank VDT operators. Univariate linear regression models reveal that their mean WAI score is inversely associated with gender, age, dependent family members, and a part-time job, whereas it is directly associated with the educational level and physical activity. In addition, multivariate analysis shows that their mean WAI score is inversely associated with age and a part-time job, but was directly associated with the educational level, the marital status, and physical activity. Overall, VDT operators working in Italian banks display high WA even though this latter tends to decline with aging. In light of the progressive aging of the workforce in Italy, our results provide the rationale for the design of interventions aimed to mitigate the detrimental effects of aging on WA of bank VDT operators.

## 1. Introduction

In the last few decades, advances in technology have led to the widespread use of computers in a variety of work environments [1,2]. Computers have become key tools in virtually every aspect of work, from managerial activities to document preparation and electronic communication. According to a study by Dangolani [3], information technology (IT) in the workplace saves labor time and cost by optimizing network operations.

The banking sector, among others, has been radically changed by the organizational and technological revolution brought by digitalization and new technologies [2,4], which experiences increased workplace flexibility, intensification of work rhythms, and overall redefinition of traditional work tasks and banking activities. As a result, banks have had to completely rethink their organization, strategies, and customer relationship management [4].

The psychosocial conditions of bank operators are characterized by high levels of job strain, scant social support, and extensive effort resulting in low rewards [5], with detrimental consequences on the psychological health of workers [6,7,8]. Due to the advent of digitalization, bank computer operators often have to spend many hours a day working at video display terminals (VDTs), which expose them to a wide range of physical and psychological risk factors. In addition, sitting at the VDT in work stations lacking ergonomic design causes workers to adopt awkward postures, using fingers repetitively and experience visual fatigue [2], which are conditions often exacerbated by poorly usable VDT software [8]. As a result, these computer operators may experience insomnia [9], computer vision syndrome, asthenopia [10], tension headache, lower back pain, and other musculoskeletal disorders [11,12,13], psychological disorders [14,15,16], and stress [17,18], with the latter having grown to critical levels in recent years [8]. 

Due to the previously mentioned issues, a rising number of companies have started to pay close attention to both the ergonomics of VDT workstations (i.e., chair, keyboard, monitor, mouse, footrest, and light). Such ergonomic regulations are laid out in article 173, paragraph 1, letter b) of Legislative Decree 81/2008—and training of VDT operators [11]. However, in spite of all these ongoing efforts, recent studies have shown that working at VDTs still exerts a negative impact on the ability to work of bank employees [17,18,19,20,21,22].

### 1.1. Work Ability

In the 1980s, the Finnish Institute of Occupational Health (FIOH) first introduced the concept of work ability (WA) to ensure proper balance among functional capacities, resources, competences, and job demands. In a nutshell, WA defines the physical and intellectual resources that workers can rely on so as to cope with the emotional, cognitive, and physical demands posed by their work [23]. In the 1990s, the FIOH also created the Work Ability Index (WAI) questionnaire, which consists of seven items that can provide a measure of the subject’s ability to work [24].

Several studies have subsequently shown that aging is negatively associated with WA [25,26,27,28] mostly due to the rising imbalance between the decreasing physical and cognitive resources of older workers and the increasing demands of their jobs [29]. The vast majority of work-related tasks and their organizational contexts are not aligned with age-related changes in workers. In this scenario, it is quite clear that WA is set to become an even more important work-related issue in the years to come since poor WA is thought to increase the number of sickness absences, early retirements, and disability pensions in addition to being the main culprit for intention to leave a job, for stress, and for depression among workers [28,30,31,32]. 

Besides aging, other factors such as gender [26], educational level [18,33,34], smoking [35], and domestic work [36] appear to be negatively associated with WA, whereas resources such as job autonomy and meaningfulness of work have been shown to positively influence WA [37,38].

Due to the progressive aging of the workforce in recent years, there has been a widespread increase in the number of studies focusing on workers’ WA. However, there is only limited research on WA of VDT operators working in the bank system [17,18,19,20,21,22].

### 1.2. Aim of the Study

In light of the WA model and recent related literature, we aimed to determine whether socio-demographic (e.g., gender, age, educational level, marital status, offspring, and dependent family members), lifestyle (e.g., physical activity, body mass index (BMI), and smoking status), and occupational (e.g., full-time employment or part-time employment) characteristics correlate with WA. We considered “offspring” and “dependent family members” as two different variables. “Offspring” specifically indicated sons and daughters who were clearly dependent family members, while “dependent family members” indicated adults who needed care. Often the term refers to a spouse or elderly parents who are not independent due to illness.

Given the Italian socio-demographic context, which, according to a recent survey by the Italian National Institute of Statistics, has become one of the oldest countries in the world, which is second only to Japan. The assessment of the impact of aging and other variables on WA of Italian workers, in particular bank VDT operators, represents an urgent unmet medical need. This is particularly relevant in light of the fact that previous literature has mainly focused on WA of workers performing physically or emotionally demanding tasks [32,33,34], while only a few studies have addressed WA in workers carrying out cognitively demanding tasks in poor ergonomic conditions, such as bank VDT operators.

## 2. Materials and Methods 

### 2.1. Data Collection and Work Ability Index (WAI) Assessment

In 2017, the researchers signed an agreement between the Department of Public Health and Pediatrics of the University of Turin and the National Institute for Insurance against Accidents at Work, INAIL (ID 44/2016, resolution of 31/05/2017). The agreement provided for the protection of personal data, pursuant to the Italian Legislative Decree 196/2003, as well as the use of the data collected only for the purpose of achieving the research objectives. Data collection was carried out within the health surveillance activity, which was executed pursuant to the Italian Legislative Decree 81/2008. We conducted a cross-sectional study on 8684 workers employed at an Italian banking group. This group operated throughout Italy, with the study participants working in different areas of the Country, including Northern (71.8%), Central (14.1%), and Southern (14.2%) Italy, as well as the islands of Sicily and Sardinia. We approached workers during standard medical check-ups and collected information through their medical records on socio-demographic factors (e.g., sex, age, education, working area, place of residence, marital status, number of children, etc.), anthropometric measures (e.g., weight and height), lifestyles (e.g., leisure-time physical activity, smoking and alcohol drinking habits, coffee consumption, etc.), personal history of disease, drug use, presence of dependent family members, job characteristics (e.g., type of job/task, part-time, commuting, type of computer used, display size, use of telephone and headphone, etc.), and, for women, reproductive factors (e.g., parity, age at menarche, age at menopause, etc.). Medical records also included other relevant information that emerged during the visits. All study participants signed an informed consent form in compliance with privacy laws in force in Italy. During standard visits, trained occupational physicians administered a validated questionnaire to assess WAI. As previously mentioned, the WAI questionnaire was developed in the 1990s by the FIOH [24]. It contains seven items, with each item accounting for a partial score that contributes to the overall WA score, ranging from 7 to 49. The seven items measure the following variables: (1) current WA compared with lifetime best (range: 1–10), (2) WA in relation to mental and physical demands of the job (range: 2–10), (3) number of current diseases diagnosed by a physician (range: 1–7), (4) estimated work impairment due to diseases (range: 1–6), (5) sick leave during the past 12 months (range: 1–5), (6) self-prognosis of WA for the next 2 years (scores: 1,4, or 7), and (7) mental resources (range: 1–4). The overall score refers to four categories of scores: 7–27 points (bad WA) to restore work ability, 28–36 points (moderate WA) to improve work ability, 37–43 points (good WA) to support work ability, and 44–49 points (very good WA) to support work ability.

We began to administer the questionnaires in late June 2017 and collected WAI data relative to 2835 workers. Workers who refused to provide information on their WAI were less than 5%. Among the workers who gave information on WAI, the eligible bank VDT operators were a total of 2077. They were all aged 20 years or older (mean age = 51 years, standard deviation (SD) = 8.9 years), and 1092 (52.6%) were men (Table 1).

### 2.2. Statistical Analysis

To assess the correlation between variables, the Spearman’s coefficient was used (Table 2). The relationship and 95% confidence interval (95% CI) between WAI and selected factors were estimated by a multivariate linear regression model. The final model included items for sex, age (in continuous), education level (low, medium, or high), marital status (single or married), physical activity (none, occasional, once per week or more than twice per week), body mass index (BMI, kg/m^2^, in continuous), smoking habits (never, used to smoke, or currently smoking), offspring, dependent family members, commuting, and part-time employment. Terms for drinking habits, coffee consumption, working area, place of residence, and other employment characteristics did not modify significantly the β estimates of the other factors considered. Thus, these terms were not included in the final model. All analyses were performed using SAS software, version 9.4 (SAS Institute, Inc. Cary, NC, USA).

## 3. Results

The educational level of most study participants ranged from medium to high (Table 1) where 59.5% of them had a secondary school diploma, while 38.2% had a bachelor or master degree, respectively. More than two thirds were married (69.0%), and 68.8% had at least one child. Half the participants did some physical activity during their leisure-time, and 25.4% of them currently smoked or had smoked in the past (17.5% and 7.9%, respectively). One in four participants were commuters, and 17.1% of them worked part-time for the bank. The mean WAI score for the participants was 44.3 (SD = 3.0).

A strong and significant inverse association was observed in the univariate model between WAI and age with β = −0.072 for a one-year increment, 95% CI: −0.086, −0.058 (Table 3). WAI was also significantly lower in the following worker groups: women (β = −0.266, 95% CI: −0.528, −0.003), divorced or widowed subjects (β = −0.504, 95% CI: −1.002, −0.005, β = −1.174, 95% CI: −2.313, −0.036, respectively), subjects who had at least one dependent family member (β = −0.798, 95% CI: −1.551, −0.046) and part-time workers (β = −0.767, 95% CI: −1.113, −0.420). WAI was inversely associated with BMI, smoking habits, and offspring, albeit not statistically significant (Table 3). In contrast, WAI was directly associated with the educational level (β for medium level = 0.868, 95% CI: −0.001, 1.738, and β for a high level = 1.757, 95% CI: 0.878, 2.635) and physical activity (β for occasional or 1 time/week = 0.706, 95% CI: 0.355, 1.057, and β for ≥2 times/week = 0.774, 95% CI: 0.477, 1.072). Commuting increased WAI, albeit not to a statistically significant level. After mutual adjustment, age (multivariate analysis: β = −0.067 for a one-year increment, 95% CI: −0.083, −0.050) and part-time job (β = −0.761, 95% CI: −1.134, −0.389) still showed a significant inverse association with WAI, whereas the education level (β for medium level = 0.855, 95% CI: −0.008, 1.719 and β for high level = 1.381, 95% CI: 0.504, 2.257) and physical activity (β for occasional or one time/week = 0.491, 95% CI: 0.143, 0.838, and β for ≥2 times/week = 0.504, 95% CI: 0.206, 0.802) displayed a significant direct association (Table 2).

**Figure 1:**
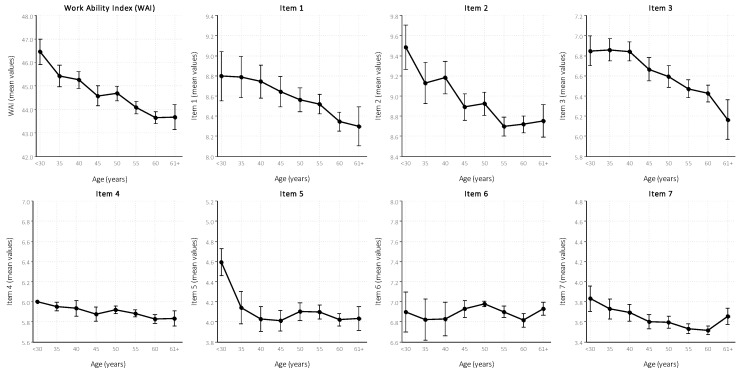
Mean and standard deviation (sd) of the work ability index (WAI) and its component, according to age groups for 2077 visual display unit (VDU) operators. Italy, 2017.Item 1: current WA compared with lifetime best. Item 2: WA in relation to mental and physical demands of the job. Item 3: number of current diseases diagnosed by a physician. Item 4: estimated work impairment due to diseases. Item 5 sick leave during the past 12 months. Item 6: self-prognosis of WA for the next two years. Item 7: mental resources.

With regard to the age, the mean WAI score ranged from 46.5 for workers aged <30 years to 43.7 for workers aged ≥61 years. Lastly, single WAI items reflected similar trends according to age, except for certain casual fluctuation (Figure 1).

## 4. Discussion

Our study participants (i.e., bank VDT operators) show a mean WAI of 44.3, which, according to the four WAI categories, is indicative of excellent WA. This is in good agreement with a previous study by Zgombić [17] recording a mean WAI score of 43.1 among bank workers, which increased to 44.1 in bank workers not interacting with customers. Our findings are also in line with two surveys among VDT workers conducted by Seibt et al. [18] and Costa et al. [19]. These works reported a mean WAI score of 41 and 40.5, respectively. Similarly, a study on bank workers by Saremi et al. [22] obtained a WAI score of 40.4. 

On the other hand, our mean WAI score is significantly higher than that reported by Guidi et al. [20], who recorded a mean WAI of 38.1 in a population of Italian bank workers. Although the sample populations of these two studies are of comparable size and background, it is noteworthy to point out that the mean age of bankers enrolled in the study by Guidi et al. is five years more than that of our study (51.0 and 45.6 years, respectively). Given that WA decreases in aging workers, we would expect to obtain a similar WAI score had we enrolled older bank operators in our study. Moreover, Guidi et al. reported a more pronounced variability in the WAI score (SD = 6.27) most likely due to the presence of various working tasks not involving VDTs and/or different modes of WAI questionnaire administration (i.e., self-administered versus interview-administered). Lastly, our findings are discordant with those reported by an Iranian study [21], where the recorded mean WAI score was 38.5. This discrepancy may be attributed to the different sample populations analyzed in these studies. Of note, both studies show similar patterns of single WAI items.

In good agreement with previous data, our findings indicate that WAI correlates with multiple factors, especially socio-demographic and occupational characteristics. In particular, this study demonstrates that age has a substantial detrimental effect on WA [25,27,28,35,36,37,38,39,40,41], in terms of both the index as a whole and each single item (Figure 1), as shown previously. This reduction of WA in older workers is likely due to the fact that individual resources tend to decrease with age, particularly physical strength and certain cognitive abilities (e.g., precision and speed of perception), while job demands remain the same or rather increase over time [29]. 

Another negative correlate for lower WA is part-time employment. More women than men have part-time contracts (32.5% vs. 3.3%, respectively). WAI among women is generally lower than that observed in men [26], likely due to the fact that women are often responsible for both work and household duties [36]. Fittingly, our univariate models show a negative correlation between being female and having dependent family members in terms of WA, which supports the negative impact that household work has on WAI. Surprisingly, the same association was not observed when the offspring variable was considered. In this regard, stratified analysis failed to reveal diversified patterns concerning the relationship between WAI and offspring, according to sex, which leaves this issue unclear. 

Being divorced or widowed negatively correlates with WA, which highlights the fact that having family support can increase WA [42]. However, it should be pointed out that that the number of widowers enrolled in our study was probably too low to draw final conclusions.

In keeping with previous studies [18,33,34], we show that a higher educational level is a protective factor for WA, likely due to the fact that workers who have higher educational levels generally have better job skills and opportunities and higher job satisfaction, as described previously [33,43]. 

Another positive correlate for higher WA is physical activity during leisure time. In particular, physical exercise positively correlates with WAI, especially among workers who partake in intense exercise more than twice a week. Consistent with our findings, others have shown how important the lifestyle, especially physical activity, can be in maintaining good physical health and high WA [21,34,44,45]. Regular exercise can increase energy levels, reduce body fat, and maintain aerobic capacity while improving self-esteem and self-perception of one’s health condition. Furthermore, according to Firoozeh et al. [44], physical exercise can improve the body’s physiological functions and reduce the incidence of chronic diseases and morbidities, which are conditions investigated by the third item of the WAI. As a result, physical activity is protective in the maintenance of WA, especially for workers who perform sedentary tasks. In this regard, Nawrocka et al. [45] have proposed that physical activity during leisure time can compensate for the time spent by white-collar workers sitting at their desks.

Although previous studies have shown that BMI [27,28,35], smoking [35,44], and commuting negatively influence WAI, we did not find these variables to be particularly relevant in our analysis. This may be, in part, explained by the different demands of the jobs in question. In our study, we investigated bank VDT operators who generally perform sedentary tasks that do not require physical performance. In this regard, being overweight or having reduced aerobic capacity as a result of smoking might not compromise the ability to work as a VDT operator. Moreover, the smoking prevalence of participants in this study was lower than that of the general Italian population. Thus, the specific employment sector under consideration for this study and/or the high educational level of participants could, in part, account for the discrepancies from the previously mentioned studies.

The cross-sectional design is one of the limitations of the present study. Although the cross-sectional study design is suitable for estimating WAI prevalence among bank VDT operators, it fails to estimate the causal relationship between WAI and the factors considered. However, it is highly unlikely that WAI would have correlated with the majority of those factors except for certain modifiable lifestyle factors such as smoking, physical activity, and BMI. 

The strengths of this study comprise the large sample size and the almost complete participation of the study subjects (≥95%). 

The main novelty of our study is that it identifies the correlates of work ability for bank workers in Italy. This is a very important aspect in light of the increasing aging of the Italian workforce and the consequent impairment of WA. Lastly, our study brings new knowledge about the bank workplace, which has previously received scant attention [46].

## 5. Conclusions

In view of the general aging of workers, especially in Italy, which inevitably leads to a decrease in WAI scores, we must promptly act on those variables that can slow down or revert this process, especially in work environments that have only been marginally investigated, such as the banking sector. In this process, it is crucial to involve VDT operators since they currently make up a large majority of the labor market, with their number expected to steadily grow in the future. 

The results of this study appear to indicate that VDT operators could preserve their WA by adopting a healthy lifestyle that supports the dimensions by positively affecting the WAI. In addition, our findings suggest that organizational and ergonomic interventions put in place by the employer could mitigate the negative effects of aging on WA. This is best exemplified by the car maker BMW, which has recently built an innovative plant specifically designed for older workers thanks to the introduction of a total of 70 improvements. Some of these improvements consisted in installing special chairs at several workstations to allow workers to work while sitting down and also relax for short periods of time during their breaks. BMW has also implemented a system of job rotation across their workstations during shifts in order to balance the load on the workers’ bodies. These changes have increased the productivity by 7% in one year [47]. Furthermore, the promotion of healthy lifestyles could also preserve and ameliorate WA [48].

For young workers, these solutions should be regarded as a form of primary prevention, while, for older workers, they should be considered as a form of secondary or tertiary prevention. Lastly, our findings call for increased efforts to strengthen welfare policies aimed to support women in maintaining their work-life balance (e.g., opening company nurseries or infant schools) and supporting older workers in the management of elderly relatives.

## Figures and Tables

**Table 1:** Distribution of 2077 visual display unit (VDU) operators, according to socio-demographics characteristics, body mass index (BMI), physical activity, smoking status, work ability index (WAI), and other selected variables. Italy, 2017.

Variables	*n* or Mean; % or SD
**Sex (*n*; %)**	
Men	1092 (52.6)
Women	985 (47.4)
**Age: years (mean; SD)**	51.0 (8.9)
**Education level ^a^ (*n*; %)**	
Low	48 (2.3)
Medium	1235 (59.5)
High	794 (38.2)
**Marital status (*n*; %)**	
Married	1433 (69.0)
Single	437 (21.0)
Divorced	159 (7.7)
Widow	28 (1.4)
Missing	20 (1.0)
**Physical activity: frequency (*n*; %)**	
None	1039 (50.0)
Occasional or 1 time/week	394 (19.0)
≥2 times/week	644 (31.0)
**Body mass index—BMI: kg/m^2^ (mean; SD)**	24.5 (10.9)
**Smoking status (*n*; %)**	
Never	1551 (74.7)
Former	163 (7.9)
Current	363 (17.5)
**Offspring (*n*; %)**	
No	649 (31.3)
Yes	1428 (68.8)
**Dependent family members (*n*; %)**	
No	2012 (96.9)
Yes	65 (3.1)
**Commuter (*n*; %)**	
No	1538 (74.1)
Yes	539 (25.9)
**Part-time job (*n*; %)**	
No	1721 (82.9)
Yes	356 (17.1)
**Work ability index—WAI (mean; SD)**	44.3 (3.0)

^a^ Low education level includes primary and secondary schools, medium education level includes high and professional schools, high education level includes bachelor and master degrees.

**Table 2:** Correlation between variables selected for 2077 visual display unit (VDU) operators. Italy, 2017.

Variables	Variables										
	Sex	Age	Education Level	Marital Status	Physical Activity	BMI	Smoking Status	Offspring	Dependent Family Member	Commuter	Part-Time Job
Sex	-										
Age	−0.16	-									
Education level	0.07	−0.26	-								
Marital status ^a^	0.01	0.18	−0.03	-							
Physical activity	−0.07	−0.15	0.10	−0.04	-						
Body mass index—BMI	−0.39	0.22	−0.16	0.07	−0.13	-					
Smoking status	−0.08	0.05	−0.06	−0.09	−0.07	0.09	-				
Offspring	0.05	0.20	−0.09	0.51	−0.07	0.09	−0.05	-			
Dependent family members	0.03	0.03	−0.01	0.05	−0.06	−0.02	0.03	0.06	-		
Commuter	−0.07	0.01	−0.01	−0.02	−0.06	0.05	−0.01	0.02	−0.05	-	
Part-time job	0.39	−0.08	0.01	0.11	−0.03	−0.17	−0.04	0.18	0.04	−0.09	-
Work ability index—WAI	−0.06	−0.21	0.16	−0.01	0.11	−0.05	0.01	−0.04	−0.05	0.03	−0.11

^a^ Analysis conducted on 2057 subjects because of missing values.

**Table 3:** Univariate and multivariate linear regression models for work ability index (WAI), according to socio-demographics characteristics, body mass index (BMI), physical activity, smoking status, and other selected variables. Italy, 2017.

Variables	Univariate		Multivariate	
	β	(95% CI)	β	(95% CI)
**Sex**				
Men	Ref.	−	Ref.	−
Women	−0.266	(−0.528, −0.003)	−0.187	(−0.472, 0.010)
**Age: years**	−0.072	(−0.086, −0.058)	−0.067	(−0.083, −0.050)
**Education level ^a^**				
Low	Ref.	−	Ref.	−
Medium	0.868	(−0.001, 1.738)	0.855	(−0.008, 1.719)
High	1.757	(0.878, 2.635)	1.381	(0.504, 2.257)
**Marital status ^b^**				
Married	Ref.	−	Ref.	−
Single	0.173	(−0.153, 0.499)	−0.326	(−0.737, 0.084)
Divorced	−0.504	(−1.002, −0.005)	−0.352	(−0.840, 0.136)
Widowed	−1.174	(−2.313, −0.036)	−0.509	(−1.621, 0.603)
**Physical activity: frequency**				
None	Ref.	−	Ref.	−
Occasional or 1 time/week	0.706	(0.355, 1.057)	0.491	(0.143, 0.838)
≥2 times/week	0.774	(0.477, 1.072)	0.504	(0.206, 0.802)
**Body mass index—BMI: kg/m^2^**	−0.005	(−0.017, 0.007)	−0.005	(−0.017, 0.006)
**Smoking status**				
Never	Ref.	−	Ref.	−
Former	−0.011	(−0.503, 0.481)	0.200	(−0.282, 0.681)
Current	−0.064	(−0.413, 0.285)	0.058	(−0.288, 0.403)
**Offspring**				
No	Ref.	−	Ref.	−
Yes	−0.188	(−0.471, 0.095)	0.240	(−0.112, 0.593)
**Dependent family members**				
No	Ref.	−	Ref.	−
Yes	−0.798	(−1.551, −0.046)	−0.637	(−1.375, 0.101)
**Commuter**				
No	Ref.	−	Ref.	−
Yes	0.204	(−0.095, 0.503)	0.155	(−0.138, 0.448)
**Part-time job**				
No	Ref.	−	Ref.	−
Yes	−0.767	(−1.113, −0.420)	−0.761	(−1.134, −0.389)

^a^ Low education level includes primary and secondary schools, medium education level includes high and professional schools, high education level includes bachelor and master degrees. ^b^ Analysis conducted on 2057 subjects because of missing values.
